# Impact of training of teachers on their ability, skills, and confidence to teach HIV/AIDS in classroom: a qualitative assessment

**DOI:** 10.1186/1471-2458-13-990

**Published:** 2013-10-21

**Authors:** Haribondhu Sarma, Mohammad Ashraful Islam, Rukhsana Gazi

**Affiliations:** 1 Centre for Nutrition and Food Security, icddr,b, Mohakhali, Dhaka 1212, Bangladesh; 2 Centre for Equity and Health Systems, icddr,b, Mohakhali, Dhaka 1212, Bangladesh

**Keywords:** HIV/AIDS education, Teachers training, Qualitative method, Bangladesh

## Abstract

**Background:**

Considering the significant impact of school-based HIV/AIDS education, in 2007, a curriculum on HIV/AIDS was incorporated in the national curriculum for high school students of Bangladesh through the Government’s HIV-prevention program. Based on the curriculum, an intervention was designed to train teachers responsible for teaching HIV/AIDS in classes.

**Methods:**

In-depth interviews were conducted with teachers to understand their ability, skills, and confidence in conducting HIV/AIDS classes. Focus-group discussions (FGDs) were conducted with students who participated in HIV/AIDS classes. HIV/AIDS classes were also observed in randomly-selected schools. Thematic assessment was made to analyze data.

**Results:**

The findings showed that the trained teachers were more comfortable in using interactive teaching methods and in explaining sensitive issues to their students in HIV/AIDS classes. They were also competent in using interactive teaching methods and could ensure the participation of students in HIV/AIDS classes.

**Conclusions:**

The findings suggest that cascading training may be scaled up as it helped increase ability, skills, and confidence of teachers to successfully conduct HIV/AIDS classes.

## Background

School-based programs offer opportunities to encourage adolescents and young people to delay the onset of sexual activity, promote safe sex behavior using a condom, and increase contraceptive-use after sexual initiation [1]. Results of most evaluation studies on curriculum-based HIV and sex education showed the impact that has positive changes in knowledge, attitudes, and reported behaviors of students [1-5]. Results of a study in China, suggest that intervention on HIV/AIDS education significantly (p < 0.001) increases knowledge of subjects, changes their attitudes positively (p < 0.001), and improves their protection self-efficacy (p < 0.001) [6]. However, a review study contradicts with earlier findings; despite the positive impact of school-based HIV/AIDS education on knowledge and attitudes, it has little impact on sexual behaviors of subjects [7].

Results of studies also suggest that, to have a substantial impact of HIV/AIDS-education programs, teachers or educators should be adequately trained before involving them in any HIV/AIDS-prevention program in a school setting [8-12]. A qualitative study in South Africa concluded that teaching about sexuality was perceived to be challenging in terms of language and communication norms, and teaching about HIV/AIDS was perceived as challenging because teachers often need to convince students about the reality of AIDS [13]. In 2010, a report concluded that there is a general consensus that more work for teacher-trainees is required and that without preparing teachers to work in this area, there is a limit to what can be achieved [14]. Moreover, training on HIV/AIDS education is facilitated educators to breaking the silence and reducing stigma while, at the same time, equipping them to provide care and support for infected and affected learners and colleagues [15]. Findings of a rapid assessment in Bangladesh showed that trained teachers were more likely to participate in classroom teaching of HIV/AIDS [16]. Despite this positive impact, almost half of trained teachers in the intervention area faced some difficulties in teaching the HIV/AIDS course, particularly during discussion on the transmission and prevention of HIV/AIDS and sex-related issues. Moreover, in the intervention area, over 50% of teachers did not use any interactive teaching method [16].

Implementing HIV/AIDS education through the national curriculum is a challenging task, especially in developing cultural-sensitive curriculum and readiness of teachers in disseminating HIV/AIDS information to their pupils. In Bangladesh, HIV/AIDS is a sensitive issue in the sociocultural-religious context due to its transmission primarily through sexual contact. It may, thus, be considered too controversial to be taught in a school setting [17]. Besides, incorporating HIV/AIDS in the national curriculum is itself a policy issue. Since education is the national concern, strong governmental commitment to the mainstream education sector is, thus, needed in terms of both planning and advocacy as a channel of disseminating HIV/AIDS-related information [17].

In 2004, the Government of Bangladesh undertook a project titled “Prevention of HIV and AIDS among young people in Bangladesh”. One of its components of the project implemented in two phases was “Integrating HIV and AIDS information into the secondary school (high school) and college curriculum as well as dissemination of developed materials”. This component had two major segments: (a) Development of curriculum and texts for class Six-Twelve and (b) Institutional capacity-building through a nationwide teachers’ training program. PIACT Bangladesh, a non-profit, non-governmental organization, is responsible for implementing the training program. A curriculum on HIV/AIDS and texts for class Six-Twelve were developed following a participatory, bottom-up approach based on public-private partnership.

For institutional capacity-building, PIACT Bangladesh developed a cascade training program in which master trainers were trained first to imparting training to core trainers, who, in turn, trained subject teachers (responsible for teaching science or social science) at the institution level. For three-day master trainers’ training held at the district level, two teachers from a college, one teacher from an alim madrasha, and the Upazila Secondary Education Officer from each upazila (subdistrict) were selected. Besides, four master trainers from each upazila in a district were also selected. These master trainers trained core trainers drawn from secondary and higher secondary schools, including dakhil and alim madrashas. Two teachers, including the head teacher/principal, attended a two-day core training program held at the upazila level. Trained in batches, each batch consisted generally of 30 teachers for the core trainers’ training program. All the core trainers were responsible for training subject teachers in their respective schools/colleges and madrashas. On average, five subject teachers from each school received training for one and a half days. The core trainers also organized a half-day orientation session with the School/Madrasha Managing Committee to create a supportive environment for teaching the subject in and around the institution.

The broad objective of training was to develop skills and knowledge of teachers so that they can appropriately communicate HIV/AIDS-related messages in the classroom setting. Training sessions were focused on providing appropriate information on HIV/AIDS and on the evolving ways for using interactive methods to teach the subject; for example, how to organize group discussions and puzzle games and how to encourage active participation of students using role-play method.

The present study was conducted to understand the effects of teachers’ training, covering a curriculum-based HIV/AIDS-education program, on ability, skills, and confidence of teachers to teach HIV/AIDS in classes.

## Methods

### Study background

The study was a follow-up assessment of a previously-conducted rapid assessment of a teachers’ training program [16]. The previous assessment was a cross-sectional quantitative survey and described the role of teachers’ training in providing HIV/AIDS education in classes. Since the earlier study used a self-administered survey questionnaire, an uncommon technique in Bangladesh, stakeholders of HIV/AIDS-education program raised concerns about the validity of data during dissemination-seminar of the study. Therefore, the present study was conducted to illuminate subjective experiences of teachers about their ability, skills, and confidence in conducting HIV/AIDS classes with students. The use of qualitative approach is not new to evaluate HIV/AIDS or sexual health programs. The earlier studies also used qualitative methods to assess the program outcomes or impacts on knowledge, attitudes, beliefs and behaviors of teachers/educators [13,18-20].

### Study design

The present study used qualitative techniques for data-collection. To triangulate findings, the study used multiple participant-groups as sources of information and also used various qualitative techniques. The study compared findings from teachers who received training on HIV/AIDS with findings from teachers who did not receive such training.

### Study sites

The study was conducted in two randomly-selected districts from a list of districts in Chittagong division of Bangladesh. One of the two districts was selected from the list of districts where subject teachers of those districts received training through the PIACT’s cascading training program at least a year before data-collection, and the other one was selected from the list of districts where no such training was held. From each district, three upazilas (each upazila having a population of 200,000-300,000) were randomly selected. While selecting an upazila, we considered similar upazilas from each district; however, we did exclude all Sadar (urban) upazilas due to sociocultural and economic variations between Sadar upazilas and other upazilas. Similar upazilas were identified by analyzing secondary data of each upazila, such as socioeconomic status, literacy rate, number of schools/colleges/madrashas, and the geographical distance from the district headquarter. The three upazilas were randomly selected from the list of similar upazilas.

### Data-collection

Data were collected during November 2010–June 2011 by conducting in-depth interviews with subject teachers, observing HIV/AIDS classes, and conducting focus-group discussions (FGDs) with students who participated in HIV/AIDS classes. A skilled and experienced four-member field research team was formed. The team comprised a Research Officer (RO), two Senior Field Research Assistants (SFRAs), and a Field Research Assistant (FRA) of icddr,b (International Centre for Diarrhoeal Disease Research, Bangladesh) for collecting data in the field. The research team was further trained on the study contexts, qualitative data-collection techniques, and non-random sampling procedures. The Principal Investigator (the first author) monitored the overall data-collection process.

#### In-depth interview with subject teachers

To conduct in-depth interviews, a flexible semi-structured interview guideline was developed. The interview guideline covered issues, such as background of participants; their experiences in training on curriculum-based HIV/AIDS education; participation in teaching of HIV/AIDS in classes; contents of text relating to HIV/AIDS taught by teachers; mode of teaching method used while educating students about HIV/AIDS; ability, skills, confidence, and experience of teachers in using interactive teaching method; how comfortable are they in discussing sensitive issues with students; their basic understanding of HIV/AIDS; and factors that hindered or facilitated during HIV/AIDS sessions. These were the prime topics of discussion; however, new issues were added based on new findings during initial interviews and analysis. On average, each interview lasted for 40-60 minutes.

As per the purposive criteria, subject teachers were selected from both study districts. In the non-intervention district, teachers who were conducting HIV/AIDS classes and did not receive any training on HIV/AIDS neither from the PIACT nor from any other institution were selected. In the intervention district, teachers who were trained under cascading training on the HIV/AIDS curriculum and who were conducting HIV/AIDS classes were selected.

The interviewers maintained a diary and took detailed field-notes to make results of interviews richer. A digital recorder was used for recording interviews. Immediately after interviews, the interviewers completed field-notes and transcribed the recording. After transcribing the previous interview, they proceeded for the next interview so that the experiences gathered during the previous interview help fill the gaps in the next one.

#### Observations of HIV/AIDS classes

The research team observed HIV/AIDS classes to understand ability of teachers in using interactive teaching method in the classroom setting and participation of students in classroom discussions. Since our intention was to observe natural methods of teaching (what was actually happening) in HIV/AIDS classes and to minimize bias, the respective teacher of the selected class was not shared beforehand about observation, except the headmaster of the respective school. For documenting information, an observation checklist (including closed- and open-ended items) was used. The observation checklist covered the teacher's clarity and understanding of the unit objectives; ability to encourage participation of students in the learning process; receptiveness to feedback from students; ability to simplify and explain subject matter; and ability to create a comfortable classroom environment. Besides completing the observation checklist, the observers also maintained notes and a diary; after each observation, a detailed report was prepared.

#### FGDs with students

The research team conducted FGDs with students who participated in HIV/AIDS classes to understand the level of their participation in a class and to assess ability of teachers to use interactive method in teaching in HIV/AIDS classes. A semi-structured guideline was used to conduct FGDs, which included practical issues relating to HIV/AIDS rather than textual information (e.g. knowledge); for example, students were asked to describe details of the HIV/AIDS class, including total class time, issues/content discussed, mode of teaching method used, and their participation in discussions, and finally, they were asked to comment on ability and comfortableness of teachers in using teaching methods. Since the FGDs took place immediately after an HIV/AIDS class was over, the research team reached the respective school immediately before the class time and took permission from the headmaster of the school for conducting FGDs. The RO of each team worked as a moderator/facilitator, and the SFRA assisted him and took detailed notes on discussions and observations (e.g. body language of participants). A voice recorder was used for recoding the whole discussion. After each FGD, the research team transcribed the recorded data, and then a detailed report was prepared incorporating all notes and transcripts for each FGD. On average, each FGD lasted for 60-90 minutes.

### Analysis of data

Analysis of qualitative data was begun with first field activities (such as in-depth interview, observations, and FGDs). This analysis guided the improvement of quality of data as the study proceeded. The investigators regularly reviewed (initially daily) the field-notes and the diary with the research team. The interviewers met the Principal Investigator at least once a week to review results of their activity sessions/interviews and to determine the best practices for further activity sessions/interviews, note-taking, and areas for questioning that should be pursued or which are not effective. From the beginning, thematic analysis was done. Themes were identified based on the existing themes and new information from the findings. The processes followed a sequence of inter-related steps recommended by Ulin *et al*. that included reading, coding, displaying, reducing, and interpreting [21]. At first, transcripts were carefully read, and then coding of data was begun. Reading and coding were initiated while data were being collected. The data-display and reduction process was conducted once all data had been collected. Even during data display and reduction, the investigators looked back through the earlier steps to refine codes, re-read texts, and revise some aspects of analysis. After reading, re-reading, and coding the text, main themes were begun to be formalized. Each theme was examined separately and fully within available data. These themes were then cross-checked with each other to generate new insights and patterns for further exploration of data. Line-by-line content, contextual and thematic analysis-strategies were performed. During analysis, diverse data were considered and were presented in the report as a typical pattern. The results were summarized and presented separately for control and intervention groups according to the objectives and contexts. Some data were presented verbatim to demonstrate complex views and ideas. Finally, to ensure quality of information, findings on similar themes were triangulated and cross-matched among findings of in-depth interviews, FGDs, and observations of different groups or individuals.

### Ethical aspects

The Institutional Review Board of icddr,b, comprising a Research Review Committee and an Ethical Review Committee, approved the study. The interviewers took written consent from the respondents before conducting in-depth interviews. For observation of HIV/AIDS classes, a written institutional consent was taken from the headmaster of the school. However, before starting observation, the observer took verbal consent from the class teacher and students. The research team took written permission from the headmaster of the school for conducting FGDs. Before initiating any discussion, the team also took written assent from all FGD participants.

## Results

In total, 36 in-depth interviews, 12 FGDs, and 12 observations were conducted in the two study sites: Lakshmipur district and Feni district. Fifty percent of the interviews, FGDs, and observations were conducted in each study district.

### Perceptions of teachers about HIV/AIDS training outcomes

#### Perceived benefits of training in preparation of lessons

The teachers perceived that the training helped them prepare class-wise lesson plans for their students. The meaning of class-wise lesson plan was that, since the students of Class Six-Eight are not matured, they would be given only a basic idea about HIV/AIDS. However, details about HIV/AIDS can be elaborated for the students of Class Nine-Ten as they are quite matured. The teachers also stated that, since the students of Class Six-Eight are in their tender age, they can become derailed or pervasive if they are given details about HIV/AIDS. They argued that, since the textbooks of Class Six-Eight contain a very basic idea about HIV/AIDS, they need not to learn more. However, when they will study in Class Nine-Ten, they can learn more details about HIV/AIDS. A subject teacher at Chagolnaiya, Feni during in-depth interview said:

“To speak the truth, if we did not get the training, it would not have been possible for us to teach HIV/AIDS in the class. After training, we have been conscious about the disease, can prepare lectures according to their needs, and can teach students easily and comfortably.”

#### Perceived benefits of training in reducing misconceptions

The teachers further stated that training helped remove many of their misconceptions relating to HIV/AIDS; for example, many teachers believed that HIV/AIDS is a contagious disease; however, after training, they understood the real facts. A subject teacher at Chagolnaiya, Feni during in-depth interview said:

“A few days ago, I heard that a person named Ali… would be tightened with a tree by confining him in a box. He would be given food using a stick.... The meaning of this is that, if he is touched, HIV can be transmitted …. After training, my misconception was removed, and I realized that it was a bitter example of stigma and discrimination!”

#### Perceived benefits of training in improvement of teaching skills

The teachers mentioned that the training helped them gather skills to explain sensitive issues relating to HIV/AIDS to their students. Before training, they felt uneasiness to explain sensitive issues but after training, their uneasiness considerably declined. They have now learnt how to explain sensitive words using synonyms, or by story-telling, or the other way. A teacher said:

Before training, the teachers would avoid to mention sensitive issues tactfully like me, saying that, since a questionnaire contains a number of questions, it is not compulsory to answer a specific question relating to HIV/AIDS …. After the training, I never avoided such topics, and now I understood how I could explain sensitive issues to students’ (A subject teacher at Fulgazi, Feni, during in-depth interview)

### Pre-training helped in using innovation in teaching methods

The non-trained teachers used lecture method while conducting HIV/AIDS classes. When the teachers were asked as to why they followed the method, they mentioned difficulties in giving a practical example concerning HIV/AIDS due to lack of knowledge on this issue. Students in FGDs stated that teachers used both lecture and question-answer methods in classes. They also mentioned that the teachers first delivered a lecture and then asked questions to them. Teachers just read out a topic that could not be heard from behind.

In classroom observations, the research team found that teachers who did not have any training on HIV/AIDS used traditional lecture method only. In lecture method, the teachers delivered lectures, and students would listen. While observing classes, it also appeared that the teacher himself was not knowledgeable about HIV/AIDS. It even appeared that the teacher was not comfortable to teach the chapter on HIV/AIDS because his confidence level was so weak that he felt nervous. Chalk and duster used by him fell down from his hand due to his excessive nervousness.

On the other hand, the trained teachers created some innovation in teaching methods. They mostly used discussion method and group-work with the help of a flip-chart, posters, and other visual aids. The teachers thought that, through group-work, students could easily discuss sensitive issues among themselves, which enable them to get an equal lesson. A teacher stated:

Students could not remember contents of a lecture if they were taught using lecture method …. In group-work, they could take notes in the notebook by discussing with their co-students … because it is equal to write once than to read three times …. (A subject teacher at Fulgazi, Feni, during in-depth interview)

In the in-depth interview, another teacher stated:

When students were taught with the help of a flip-chart, they became fascinated because they found some newness in the chart …. (A subject teacher at Fulgazi, Feni, during in-depth interview)

In the question-answer sessions, the teachers informed the students before-hand about the topic that will be discussed in the next-day class so that they can understand the issue easily and can participate in the discussion session. The teachers thought that, through this method, teacher-student interactions became closer. The student-participants in FGDs in the intervention area also stated that, in group-work, a teacher first delivered a lecture and then formed groups by giving several topics to write some points relating to the particular topic. Following this, the team leader presented some points, and other groups provided their feedback.

During observation, it was noticed that a trained teacher used hand-notes (prepared by him) and a flip-chart in the class. While observing classes, it appeared that concept of a trained teacher about HIV/AIDS was clear, and he tried to explain the concept easily and comfortably. In group-work, students were divided into groups first, and they were then told to give a name of the group by the name of a river, such as Padma, Megna, or Jamuna. Each team was then given a small chit (where the topic was written), a sign pen, and a large-size paper. Ten minutes were allocated for group-work. After completing the group-work, the team leader presented findings. Then the teacher told students to ask questions. Since the students did not ask any question, the teacher discussed issues not included in group-work**.**

### Training helped teachers to be comfortable in conducting HIV/AIDS classes

#### Comfortableness in explaining sensitive issues

The trained teachers were conscious in explaining sensitive issues in HIV/AIDS classes. They stated that, as they learnt from training, they could prepare class-specific lessons. Only basic information about HIV/AIDS was provided to students of Class Six-Eight, and some detailed information was provided to students of Class Nine and Ten. The teachers opined that students of lower classes might have been derailed if they were given details about sensitive information relating to HIV/AIDS. Synonyms were, therefore, used for explaining sensitive issues where the religious rules were emphasized. A teacher stated:

We applied a technique to explain sensitive issues because students of Class Eight are in their tender age. However, since students of Class Nine-Ten are matured, they were given class-specific detailed lessons. (A madrasha subject teacher at Fulgazi, Feni, during in-depth interview)

The student-participants in FGDs stated that their teachers laid emphasis on the religious rules to explain socially-accepted sensitive issues. When the FGD participants were asked to explain as to how their teachers discussed sensitive words in the class, one FGD participant replied:

The religious rule means not to involve in any physical relationship irrespective of marriage. If the religious rules would have been maintained, they would not go for any illegal physical relationship. 'Safe sex’ was explained as having marriage by maintaining the religious rules. (A FGD conducted at Parshuram, Feni, with students of Class Nine)

According to the non-trained teachers, explaining sensitive issues to students was embarrassing and sometimes difficult as they asked many questions that were impossible to answer. They were sometimes unaware of the appropriate answer or the way of explaining the issue. Moreover, when sensitive words were discussed in the class, students laughed and did not feel interest to learn about HIV/AIDS. The teachers just read out sensitive words without explaining these to students. They stated that some words, e.g. sperm and vaginal fluids, were difficult to explain. A teacher stated:

I neither uttered any sensitive word loudly nor did I put less emphasis on those words because students might feel shy or might put their heads down. So, I just read sensitive words just like a person reading a book …. (A teacher at Ramgonj, Lakshmipur, during in-depth interview)

Almost similar findings were revealed from FGDs with students in the area where teachers did not receive training. They stated that their teachers did not even look at them, and they just read out the text. If any student would try to ask any question, the teacher showed disinterest to respond. The teacher even sometimes acted like as if he could not hear the student’s question. During observations, the study team noticed that the teacher just read out text relating to modes of transmission and prevention while students were just listeners. When the teacher said that HIV/AIDS could be transmitted through an illegal relationship, students appeared to feel shy but the teacher did not notice it.

#### Most avoided issues in classroom discussion

The trained teachers mostly avoided the words 'condom-use’. Students stated that, while talking to them in FGDs, their teachers avoided the words 'sexual intercourse’. While observing the class, the research team noticed the same.

However, non-trained teachers found many words sensitive to them in classroom discussions. They usually avoided the words, such as sexual intercourse, condom-use, sperm, safe sex, and vaginal fluid. In FGDs, the student-participants reported that their teachers avoided to mention the words—sexual intercourse and condom-use. While observing the class, the research team as well noticed that teachers were trying to avoid these words.

#### Participation of students in HIV/AIDS class

The findings of FGDs in schools where teachers did not receive any training revealed that students did not participate spontaneously in HIV/AIDS classes. In most cases, the teachers did not encourage them to participate in the class, and they seemed to be annoyed when students asked questions. The research team observed that students were laughing in the HIV/AIDS class. It was also observed that teachers were delivering lectures in their own way but students had no concentration. Some teachers in the control area sometimes encouraged students to ask questions. Some teachers asked questions to students as a technique to keep the environment calm and quiet or to stop laughing. Students who participated in FGDs reported that their teachers did not take any initiative to encourage them to participate in discussions. During observations, the research team also noticed that the teachers taught their students in their own way. However, the trained teachers encouraged their students to participate in group-work and provided feedback during feedback sessions. Similar findings were observed during observations that teachers conducting a class through group-work and question-answer sessions. Students echoed same finding in FGDs.

#### Addressing practical issues

During in-depth interviews, teachers were asked to identify practical issues they faced while conducting HIV/AIDS classes. Teachers, irrespective of their training status, identified issues that were confounding their HIV teaching, such as class time, lack of support from the headmaster, teaching materials, and preference of teachers either for co-education or for separating boys and girls in the class, i.e. a separate class arrangement.

The allocated time for HIV/AIDS class was 35 minutes in both study areas. The control-area teachers reported that inadequate time was allocated to teach new and sensitive issues such as HIV/AIDS. They recommended increasing class time from 35 minutes to one hour. When we interviewed the head teachers, they argued that class time is a technical issue and was determined taking into consideration all possible issues, including the importance of different class-contents. They considered lesson on HIV/AIDS less important in terms of the examination context, and a class was allocated after the lunch period.

The trained teachers confirmed that allocated time for teaching HIV/AIDS was adequate. During training, the teachers learnt how to accommodate required issues within a speculated class time. They argued that class time could not be increased due to the schedule problem as it will hamper the usual schedule of other classes. They suggested that a teacher could divide the HIV/AIDS curriculum into units to teach it in one or more classes while making the schedule for term examination.

The trained teachers also reported that the head teacher instructed them to teach the chapter in allocated class time. They also added that the Managing Committee of the school, local people, and other teachers were informed about the issue, which helped them teach such sensitive topics comfortably and easily. All intervention-area teachers also reported that training material helped them teach the HIV/AIDS subject in the class. Besides, the material helped enhance their mental courage because they thought that whenever they would forget any issue, they can get help from the material. Although they preferred a separate class for girls and boys, they thought that co-education is okay.

## Discussion

Training on HIV and AIDS helped the study teachers to increase their ability and skills to prepare class-specific lessons. The trained teachers followed these curricula considering the adaptability of young students to explain sensitive issues and words to students and started the class by giving an introduction about the importance of the topic in their personal life. Lack of training for teachers was also identified as a concern in teaching sexual health programs in the school context [13,22,23] because teaching students about sex and sexuality often generates a great deal of anxiety as some teachers are frightened of encouraging sexual activity, or of parents accusing them of this, or they feel that it is inappropriate for them to teach sensitive issues to young students [23,24]. While previous literature suggested that societal attitudes affect personal willingness of teachers to discuss about the prevention of HIV/AIDS with their students and make them uneasy about potential community opposition [25].

Studies have shown that training helps improve knowledge, attitudes, and readiness of teachers to impart HIV/AIDS-prevention information to their students [13,26,27]. The findings of the present study also revealed that the study teachers had gathered comprehensive knowledge on HIV/AIDS through the cascading training program, which they did not have before. The intervention-area teachers thought that, through training, their misconceptions relating to HIV/AIDS could be reduced; their knowledge on HIV/AIDS increased; and they could acquire skills to teach sensitive issues. Previous literature correlated that teachers who participated in the HIV/AIDS-education program were happy and felt developed, and the program helped them increase their ability to teach and increase their knowledge, and the most important thing was that it opened their mind about this sensitive issue [28]. The same study also explored that teachers felt empowered in participation of HIV/AIDS-education intervention, ultimately resulting in an increased awareness about the problems [28].

In terms of teaching methods used in conducting HIV/AIDS classed, training had a greater impact which was reflected among teachers who had prior training. They used group-work and discussion method in classes in accordance with the situation or environment of the classroom setting. The non-trained teachers mostly used lecture method, which was also used in other classes. They, however, had no idea about any other methods to teach the topic perfectly. While previous studies also identified these as the key barriers to implementing HIV education, such as teachers’ knowledge gap, lack of guidelines and resources, and discomfort in using interactive teaching methods [29,30].

The findings of the present study demonstrated that the training enhanced skills and ability of teachers ensuring participation of students in classroom discussions. The trained teachers encouraged their students to participate in group-work and provided feedback to them to ensure their participation in discussions. While participation of students in HIV/AIDS classes was not spontaneous in the control area, in most cases, teachers did not encourage their students to participate in discussions.

Teaching school students about HIV/AIDS may be especially problematic in Bangladesh and other South Asian countries where HIV is mostly transmitted through heterosexual contact but where discussion of sexual issues among adults and students has been culturally restricted. Studies in other countries have identified that cultural prohibition created barriers to discussing these sensitive terms with students [25,31]. The findings of the present study revealed that the non-trained teachers just read out sensitive issues and words without explaining these to their students. While teaching, they mostly avoided the words, such as intercourse, condom-use, sperm, and vaginal fluid. However, this situation was more likely to change where teachers were trained on specific contents.

Overall, the results of the assessment revealed that the teachers’ training program had a positive impact on ability, skills, and self-confidence of teachers in conducting HIV/AIDS classes, who received training on HIV/AIDS through the cascading training process compared to teachers who did not receive it. The positive effects of the cascading training were also visible in this study when experiences of teachers who did not receive any training were considered. HIV/AIDS being a new subject contains some sensitive terms or words; therefore, the teachers should be trained on teaching methods, and at the same time, they need to prepare their students to be ready to learn such sensitive words or terms. Results of a study also suggest that teachers should be well-trained to be able to teach children and adolescents about human sexuality and prevention of HIV/AIDS comfortably and competently; otherwise, they will be in a disadvantageous position in dealing with populations at risk of HIV infection [32].

### Limitations

The present study had some limitations. The findings may not be generalized in the wider context due to its qualitative nature and approaches that applied to select teachers from different schools. The non-random recruitment and sampling of participants through a purposive sampling technique is prone to selection bias. Another potential limitation was that the study used direct observation; it is susceptible to observer-bias and observer-effect, which refer to the way in which presence of an observer in some way influences behavior of teachers and students being observed. However, to reduce such biases, we used trained and skilled research staff to conduct observations. The results at best give a comprehensive and qualitative understanding of the effects of teachers’ training on curriculum-based HIV/AIDS-education program on ability, skills, and confidence of teachers to teach HIV/AIDS in the classroom setting.

## Conclusion

Overall, the findings suggest that cascading training should be scaled up as it demonstrated to have its impact on teachers. This suggestion is supported because the training program particularly helped increase ability, skills, and confidence of teachers to conduct HIV/AIDS classes, increase their knowledge and skills to implement interactive teaching methods, and ensure participation of students in HIV/AIDS clasess.

## Competing interests

The authors declare that they have no competing interests.

## Authors’ contributions

All authors of this manuscript were the investigators of the research and participated in preparing the manuscript, from conception to finalization. HS designed the study, supervised and monitored data collection, analysis and drafted the manuscript. MAI participated in study design, data-collection and write-up. RG participated in study design, data analysis and write-up. All authors read, critically revised, and approved the final manuscript.

## Pre-publication history

The pre-publication history for this paper can be accessed here:

http://www.biomedcentral.com/1471-2458/13/990/prepub
